# Accurate Determination of the Josephson Critical Current by Lock-In Measurements

**DOI:** 10.3390/nano11082058

**Published:** 2021-08-13

**Authors:** Razmik A. Hovhannisyan, Olena M. Kapran, Taras Golod, Vladimir M. Krasnov

**Affiliations:** 1 Department of Physics, Stockholm University, AlbaNova University Center, SE-10691 Stockholm, Sweden; olena.kapran@fysik.su.se (O.M.K.); taras.golod@fysik.su.se (T.G.); 2 Moscow Institute of Physics and Technology, 9 Institutskiy per., 141700 Dolgoprudny, Russia

**Keywords:** Josephson effect, superconductivity, quantum electronics, nano-devices

## Abstract

Operation of Josephson electronics usually requires determination of the Josephson critical current $I_{c}$, which is affected both by fluctuations and measurement noise. Lock-in measurements allow obviation of 1/f noise, and therefore, provide a major advantage in terms of noise and accuracy with respect to conventional dc measurements. In this work we show both theoretically and experimentally that the $I_{c}$ can be accurately extracted using first and third harmonic lock-in measurements of junction resistance. We derived analytical expressions and verified them experimentally on nano-scale Nb–PtNi–Nb and Nb–CuNi–Nb Josephson junctions.

## 1. Introduction

A Josephson junction (JJ) is the key element of superconducting electronics [1]. The operation of a Josephson device usually involves manipulation and determination of the Josephson critical current, $I_{c}$. Conventional dc measurements of $I_{c}$ are complicated by two factors. First, $I_{c}$ in small junctions is subject to both thermal and quantum fluctuations [2,3,4,5]. The latter are particularly large in quantum devices, such as qubits, and require statistical determination of $I_{c}$ with a large number of measurements [2,5,6,7]. Fluctuations are significant, even for classical devices containing small JJs, such as sensors [8], nano-SQUIDs [9,10,11,12,13] and low-dissipation digital electronics [1,14], and for JJs used in fundamental studies of unconventional superconductors [15,16,17]. Second, dc measurements are strongly affected by the flicker 1/f noise. Fluctuations and noise together could lead to smearing of the current–voltage (*I*–*V*) characteristics of JJs [4] and make $I_{c}$ an ill-defined quantity. Lock-in measurements at high enough frequencies facilitate obviation of the 1/f noise. Simultaneously, they allow statistical averaging over an arbitrary number of periods. In recent works [18,19] it has been noticed that the magnetic field modulation of the junction lock-in resistance reflects the corresponding $I_{c}\left(H\right)$ modulation and can be used for extraction of $I_{c}$. However, such extraction requires proper mathematical justification and experimental verification, which was the main motivation for this work.

In this work we studied both theoretically and experimentally how the critical current of resistively shunted Josephson junctions (RSJ) can be deduced from lock-in measurements of ac resistance, $R_{ac}$. First we present a simple analytical solution for the relation between $I_{c}$ and different harmonics of $R_{ac}$. Next, we use expressions derived for determination of $I_{c}$ for nano-scale, proximity-coupled Nb–PtNi–Nb and Nb–CuNi–Nb JJs. We demonstrate that the formalism leads to a robust reconstruction of $I_{c}$ in a broad range of ac-current amplitudes, $I_{ac}$. We also show that, with some minor adjustments taking into account the eventual field-dependence of the normal resistance, $R_{n}\left(H\right)$, and deviations of the *I*–*V* shape from the RSJ model, the formalism can be employed for accurate determination of the $I_{c}\left(H\right)$ modulation. We conclude that it is advantageous to use both the first and the third lock-in harmonics for unambiguous determination of $I_{c}$.

## 2. Results and Discussion

### 2.1. Theoretical Analysis of the Lock-In Response in the RSJ Model

The shape of the *I*–*V* in the RSJ model is

(1) $$V=IR_{n}\sqrt{1-{\left(I_{c}/I\right)}^{2}}$$

for $I>I_{c}$ and V=0 for $I<I_{c}$. We assume that the bias is provided by the periodic ac current, $I=I_{ac}\sin\omega t$, with the period T=2π/ω and the amplitude $I_{ac}>I_{c}$. The *m*-th harmonic of the lock-in response at $\omega_{m}=m\omega$ is given by the *m*-th Fourier component:

(2) $$V_{m}=\frac{1}{T}\int_{-T/2}^{T/2}V\left(t\right)\sin\left(m\omega t\right)dt.$$

Equations (1) and (2) lead to simple expressions for lock-in harmonics of resistance, $R_{m}=V_{m}/I_{ac}$, the first three of which are:

(3) $$\begin{array}{c} \frac{R_{1}}{R_{n}}=1-{\left[\frac{I_{c}}{I_{ac}}\right]}^{2}, \end{array}$$

(4) $$\begin{array}{c} R_{2}=0, \end{array}$$

(5) $$\begin{array}{c} \frac{R_{3}}{R_{n}}={\left[\frac{I_{c}}{I_{ac}}\right]}^{4}-{\left[\frac{I_{c}}{I_{ac}}\right]}^{2}. \end{array}$$

Thus the $I_{c}$ can be deduced from either the first or the third harmonic of the lock-in resistance:

(6) $$\begin{array}{c} I_{c}\left(R_{1}\right)=I_{ac}\sqrt{1-\frac{R_{1}}{R_{n}}}, \end{array}$$

(7) $$\begin{array}{c} I_{c}\left(R_{3}\right)=\frac{I_{ac}}{\sqrt{2}}\sqrt{1-\sqrt{1+4\frac{R_{3}}{R_{n}}}} \end{array}$$

In experiments, it often happens that the *I*–*V* is asymmetric with different positive and negative critical currents, $I_{c+}\neq I_{c-}$. This is typically due to the self-field effect, or junction inhomogeneity [20,21]. In such a case, ${\left(I_{c}/I_{ac}\right)}^{k}$ (k=2,4) in Equations (3) and (5) should be replaced by the mean value $[{\left(I_{c+}/I_{ac}\right)}^{k}+{\left(I_{c-}/I_{ac}\right)}^{k}]/2$. Since now there are two unknown parameters, $I_{c+}$ and $I_{c-}$, their determination requires knowledge of both $R_{1}$ and $R_{3}$:

(8) $$\begin{array}{c} I_{c\pm }=\frac{I_{ac}}{\sqrt{2}}\sqrt{a\pm \sqrt{b-a^{2}}},\\ a=\left(1-\frac{R_{1}}{R_{n}}\right),\;b=\left(\frac{R_{3}}{R_{n}}+\frac{R_{1}}{R_{n}}-1\right). \end{array}$$

All even harmonics remain at zero, unless there is hysteresis in the *I*–*V* with retrapping current $I_{r}<I_{c}$ [5]. In this case, Equations (3) and (4) should be replaced by $R_{1}/R_{n}=\left(a_{c}^{2}+a_{r}^{2}\right)/2$ and $R_{2}/R_{n}=\left(4/3\pi \right)\left[a_{c}^{3}-a_{r}^{3}\right]$, where $a_{c,r}^{2}=1-{\left(I_{c,r}/I_{ac}\right)}^{2}$. Similarly to the asymmetric case, Equation (8), measurements of two harmonics, $R_{1,2}$, are needed for determination of the two unknown variables $I_{c}$ and $I_{r}$ in this case.

Finally, we note that the shape of the *I*–*V* may deviate from the RSJ expression, Equation (1). In general, a similar analysis can be expanded to any shape of the *I*–*V*. We do not consider this rigorously here because there is no explicit analytical solution. Instead, we propose a simple phenomenological modification of Equation (6) with an additional fitting parameter β:

(9) $$I_{c}\left(R_{1}\right)=I_{ac}{\left[1-\frac{R_{1}}{R_{n}}\right]}^{\beta },$$

with β=0.5 in the RSJ case, Equation (6).

### 2.2. Comparison with Experiment

**Figure 1:**
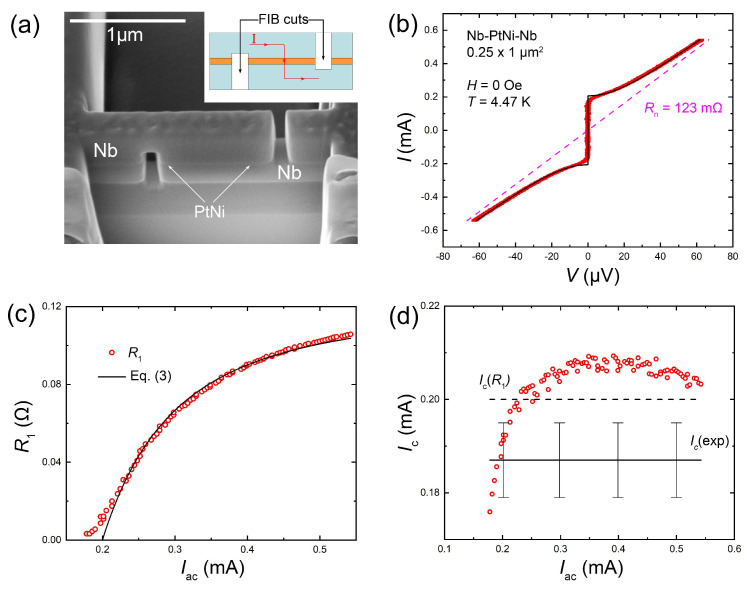
(**a**) An SEM image and a sketch of a Nb–PtNi–Nb junction. (**b**) Experimental current–voltage characteristics of the junction at H=0 and T=4.47 K (red symbols). The black line represents the RSJ fit, Equation (1). (**c**) Red circles show the dependence of the first harmonic lock-in resistance on the ac-current amplitude for H=0 and T=4.47 K. The black line shows the fit by Equation (3), using $I_{c}$ as a fitting parameter. (**d**) Red circles represent $I_{c}$ vs. $I_{ac}$ reconstructed from the data in (**c**), using Equation (6), with $R_{n}$ as a fitting parameter. The solid horizontal line represents $I_{c}\left(\exp\right)$ obtained from the *I*–*V* in (**b**) with error bars due to smearing at $I≃I_{c}$. The dashed line represents $I_{c}\left(R_{1}\right)$ obtained from the fitting by Equation (3) in (**c**). A small systematic overestimation of the reconstructed $I_{c}$ was caused by smearing of the experimental *I*–*V* by fluctuations and noise.

We present data for nano-scale, proximity-coupled junctions Nb–PtNi–Nb and Nb–CuNi–Nb. The junctions were made from trilayer films using 3D nanosculpturing via focused ion beam (FIB). Details of fabrication and junction characteristics can be found in [18,19,22]. Figure 1a shows a scanning electron microscope (SEM) image and a sketch of one of the Nb–PtNi–Nb junctions (see [22] for more details about the properties of Nb–PtNi–Nb JJs).

Figure 1b shows the *I*–*V* characteristics of a Nb–PtNi–Nb junction of area 250×1000 nm${}^{2}$ at a fixed T=4.47 K and with no applied magnetic field, H=0. Red dots represent experimental data, and a thin black line, the corresponding numerical fits using the RSJ Equation (1). It can be seen that the fit was good with the exception of the region close to $I_{c}$. The deviation may have been either due to an intrinsic difference of the *I*–*V* shape with a smoother increase of voltage at $I≃I_{c}$ than in Equation (1), or due to smearing by fluctuations and noise [4,5]. Therefore, the fit by Equation (1) yielded a somewhat overestimated value of $I_{c}\left(\mathrm{Eq}.1\right)=200\;\mu$A, which is larger than the value deduced from the experimental *I*–*V*, $I_{c}\left(\exp\right)=187\pm 8\;\mu$A, where the uncertainty was due to smearing.

Figure 1c represents the measured first harmonic resistance of this junction, $R_{1}$, as a function of $I_{ac}$ (red circles) at H=0 and T=4.47 K. Lock-in measurements were performed at f=13 Hz with the averaging time of 1 s. The black solid line was obtained from Equation (3), using $I_{c}$ as the only fitting parameter. The fit worked well with a broad range of $I_{ac}$ and yielded $I_{c}(\mathrm{Eq}.3)=200\;\mu$A. Figure 1d represents $I_{c}$ deduced from the same $R_{1}\left(I_{ac}\right)$ data with the help of Equation (6), using $R_{n}$ as the only fitting parameter. Horizontal lines show $I_{c}\left(\exp\right)$ (solid) and $I_{c}(\mathrm{Eq}.1)=I_{c}\left(\mathrm{Eq}.3\right)$ (dashed line) values. It can be seen that all methods of reconstruction of $I_{c}$ from $R_{1}$ worked well and provided $I_{c}$ values within the experimental uncertainties, marked by error bars on $I_{c}\left(\exp\right)$ in Figure 1d. From Figure 1c,d it can be seen that the reconstruction provided reliable $I_{c}$ values over a broad bias range, $1.3I_{c}<I_{ac}<2I_{c}$. Discrepancies outside this range were caused by deviations of the *I*–*V* shape from the RSJ Equation (1) due to smearing at low biases, and possibly, self-heating at large biases [5]. The independence of the $I_{c}$ extracted from the bias, $I_{ac}$, indicates the robustness of the method.

### 2.3. Reconstruction of Magnetic Field Modulation $I_{c}\left(H\right)$

Magnetic field modulation, $I_{c}\left(H\right)$, is a figure of merit for JJ quality and uniformity [20]. Measurements of $I_{c}\left(H\right)$ with integers of flux quanta in the JJ and in strong fields, when $I_{c}\left(H\right)$ becomes small, is challenging because of the enhanced susceptibility to fluctuations and noise at low Josephson energies [2]. Lock-in measurements of $I_{c}$ become particularly useful in such cases [18,19].

**Figure 2:**
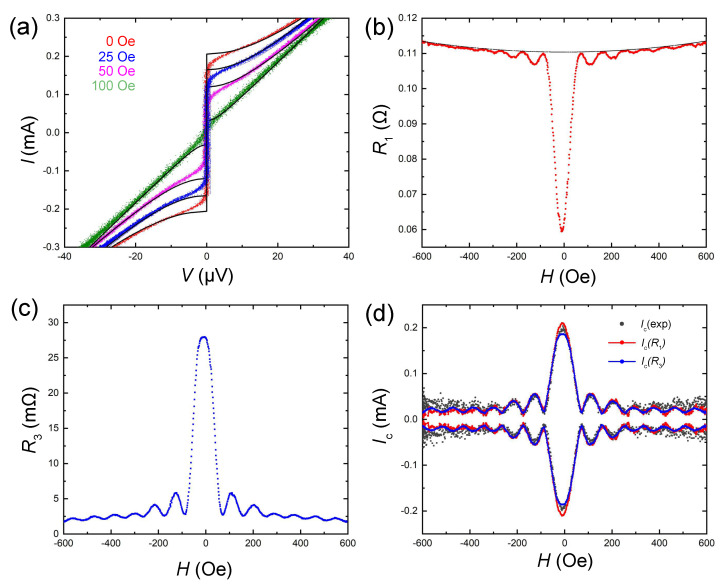
(**a**) *I*–*V* characteristics of the Nb–PtNi–Nb junction at T=4.47 K, at different in-plane magnetic fields. Black lines represent RSJ fits. (**b**,**c**) Field modulation of the first (**b**) and the third (**c**) lock-in harmonics of resistance for this junction. (**d**) Magnetic field modulation of critical currents measured experimentally, $I_{c}\left(\exp\right)$, (black symbols) and reconstructed from the first (red) and third (blue line) lock-in harmonics.

Figure 2a shows a set of *I*–*V*s for the same Nb–PtNi–Nb JJ at T=4.47 K and for different in-plane magnetic fields perpendicular to the long side of the JJ. It is clear that the $I_{c}$ is completely suppressed at H≃100 Oe. Figure 2b,c show the first and third harmonics of lock-in resistance vs. *H*, measured at a fixed $I_{ac}=315\;\mu$A. Both carry information about the Fraunhofer $I_{c}\left(H\right)$ modulation. Due to the small sizes of the JJ, the flux quantization field and the overall field range are rather large. This leads to a visible parabolic field dependence of the junction resistance $R_{n}\left(H\right)$, indicated by the black line in Figure 2b. Black dots in Figure 2d represent the magnetic field modulation of $I_{c}\left(\exp\right)$, obtained directly from the *I*–*V*s. The determination was made using a threshold voltage criterion, $V<V_{th}$. Red and blue lines represent $I_{c}\left(R_{1}\right)$ and $I_{c}\left(R_{3}\right)$ values, recalculated from the first and third lock-in harmonics, respectively, using Equations (6) and (7) with the actual $R_{n}\left(H\right)$ dependence, shown in Figure 2b. It can be seen that both modulation patterns $I_{c}\left(R_{1}\right)$ and $I_{c}\left(R_{3}\right)$ are in quantitative agreement with $I_{c}\left(\exp\right)$ within the whole range of fields. In high fields, |H|>300 Oe, modulation of $I_{c}\left(\exp\right)$ was practically unresolvable, but for $I_{c}\left(R_{1}\right)$ and $I_{c}\left(R_{3}\right)$ it can be seen clearly. Furthermore, $I_{c}\left(R_{3}\right)$ had a significantly larger signal-to-noise ratio than $I_{c}\left(R_{1}\right)$ due to less 1/f noise.

**Figure 3:**
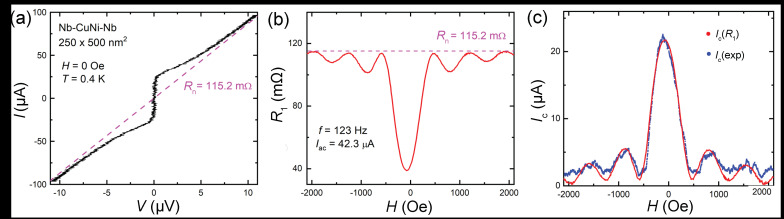
(**a**) The *I*–*V* characteristics of a Nb–CuNi–Nb junction at H=0 and T≃0.4 K. The dashed line indicates normal resistance $R_{n}=115.2\;$mΩ. (**b**) Measured field dependence of the first harmonic lock-in resistance $R_{1}$. The horizontal dashed line indicates the $R_{n}$ level. (**c**) Field modulation of the critical current, determined using *I*–*V*s (blue symbols), and recalculated with $R_{1}$ (red line) using Equation (9).

In Figure 3 we analyze data for another Nb–CuNi–Nb junction, 250×500 nm${}^{2}$ (for more details about junction properties, see [18,19]). Figure 3a shows the *I*–*V* at H=0 and T≃0.4 K. Here a deviation from the RSJ shape, Equation (1), in a form of a smoother, almost linear, deviation of *V* from zero at $I∼I_{c}$ can be seen more clearly than for the Nb–PtNi–Nb JJ, Figure 1a. Figure 3b shows field modulation (for the downward field sweep) of the first harmonic lock-in resistance measured at f=123 Hz and $I_{ac}≃42.3\;\mu$A. Figure 3c shows magnetic field modulation of the measured $I_{c}\left(\exp\right)$ (blue symbols) obtained using a threshold criterion from the *I*–*V* curves. Since the shape of the *I*–*V*s of this junction deviates from RSJ, we used the modified expression Equation (9), using β as the only fitting parameter for extraction of $I_{c}\left(R_{1}\right)$. The red line in Figure 3c demonstrates the result of this fitting with β=0.8. Apparently, it not only properly reproduced $I_{c}\left(H\right)$, but also significantly reduced noise and corrected an artifact of inaccurate dc measurements of small critical currents, $I_{c}<V_{th}/R_{n}$. Thus, the introduction of a phenomenological parameter β provided a simple way of accounting for the non-RSJ shape of the *I*–*V* curve of a junction.

Finally, we want to emphasize that the discussed method is applicable for junctions with RSJ-like *I*–*V*s, with arbitrary $I_{c}$ and $R_{n}$, at any *T*, and for any type of fluctuations (quantum or thermal). In Figure 3c the smallest reconstructed $I_{c}$ at H∼±2000 Oe is in the 100 nA range and the readout voltage $I_{c}R_{n}\;∼\;10$ nV. These are very good numbers for conventional measurements with an averaging time of 1 s and without any special precautions.

## 3. Conclusions

To summarize, we have shown that lock-in measurements can be advantageously used for accurate determination of critical currents in small Josephson junctions, for which direct dc determination of $I_{c}$ is complicated by noise and fluctuations. We have derived explicit and simple analytic expressions for the RSJ model and suggested a simple phenomenological modification for the non-RSJ case. The formalism was verified experimentally on nano-scale, proximity-coupled junctions. We conclude that it is advantageous to measure both the first and the third lock-in harmonics, which together allow robust and almost bias-independent reconstruction of the critical current. Generally it may be useful to also measure higher odd harmonics for further improvement of the proposed method. We argue that the developed technique provides a major advantage for read-outs of various superconducting devices.

## Data Availability

The data presented in this study are available on request from the corresponding author.
